# Wearing lower-body compression garment with medium pressure impaired exercise-induced performance decrement during prolonged running

**DOI:** 10.1371/journal.pone.0178620

**Published:** 2017-05-31

**Authors:** Sahiro Mizuno, Mari Arai, Fumihiko Todoko, Eri Yamada, Kazushige Goto

**Affiliations:** 1 Graduate School of Sports and Health Science, Ritsumeikan University, Kusatsu, Shiga, Japan; 2 DESCENTE Ltd., Osaka, Japan; 3 Faculty of Sports and Health Science, Ritsumeikan University, Kusatsu, Shiga, Japan; Universita degli Studi di Roma 'Foro Italico', ITALY

## Abstract

**Objective:**

To investigate the effect of wearing a lower body compression garment (CG) exerting different pressure levels during prolonged running on exercise-induced muscle damage and the inflammatory response.

**Methods:**

Eight male participants completed three exercise trials in a random order. The exercise consisted of 120 min of uphill running at 60% of VO_2_max. The exercise trials included 1) wearing a lower-body CG with 30 mmHg pressure [HIGH]; 2) wearing a lower-body CG with 15 mmHg pressure [MED]; and 3) wearing a lower-body garment with < 5 mmHg pressure [CON]. Heart rate (HR), and rate of perceived exertion for respiration and legs were monitored continuously during exercise. Time-course change in jump height was evaluated before and immediately after exercise. Blood samples were collected to determine blood glucose, lactate, serum creatine kinase, myoglobin, free fatty acids, glycerol, cortisol, and plasma interleukin-6 (IL-6) concentrations before exercise, 60 min of the 120 min exercise period, immediately after exercise, and 60 min after exercise.

**Results:**

Jump height was significantly higher immediately after the exercise in the MED trial compared with that in the HIGH trial (*P* = 0.04). Mean HR during the 120 min exercise was significantly lower in the MED trial (162 ± 4 bpm) than that in the CON trial (170 ± 4 bpm, *P* = 0.01). Plasma IL-6 concentrations increased significantly with exercise in all trials, but the area under the curve during exercise was significantly lower in the MED trial (397 ± 58 pg/ml·120 min) compared with that in the CON trial (670 ± 86 pg/ml·120 min, *P* = 0.04).

**Conclusion:**

Wearing a lower body CG exerting medium pressure (approximately 15 mmHg) significantly attenuated decrease in jump performance than that with wearing a lower body CG exerting high pressure (approximately 30 mmHg). Furthermore, exercise-induced increases in HR and the inflammatory response were significantly smaller with CG exerted 15mmHg than that with garment exerted < 5 mmHg.

## Introduction

During the past two decades, use of compression garment (CG) during exercise has been prevalent among a variety of team sport athletes as a strategy to enhance performance. Several studies have determined the beneficial effect of wearing CG on performance during exercise [1–3]. However, inconsistent results have been reported in the literature because of differences in experimental conditions (e.g., exercise duration, exercise intensity and modality, CG pressure level exerted and covered area) among studies. These differences in methodological criteria may have masked the effectiveness of CG [4,5].

To date, a number of studies focused on the effect of CG on exercise performance during running [6]. For instance, reduced energy cost of running, i.e. decrease in oxygen uptake (VO_2_) slow component [1], and increased time to exhaustion [2] were observed when wearing CG during submaximal running, whereas previous studies still contain conflicting results for benefit of wearing CG during running [7–9]. Possible explanations for these discrepancies may be due to differences in the levels of compressive pressure applied among studies [5]. Considering that higher compression (> 35 mmHg) may reduce local blood circulation around muscle [10], moderate compressive pressures (15–20 mmHg) may be appropriate for improving exercise performance. In this regard, Ali et al. [3] determined the effects of different levels of pressure exerted by CG (covered knee to ankle) on 10-km time trial performance. As a result, two different types of CG, exerting 12 or 18 mmHg pressure at the knee, significantly attenuated the exercise-induced decrease in jump performance compared with the control trial (0 mmHg), while CG with high pressure (23 mmHg at the knee) did not improve performance. Furthermore, Miyamoto et al. [11] showed that wearing CG applying 15 or 20 mmHg to thigh significantly attenuated exercise-induced increase in skeletal muscle proton transverse relaxation times, which reflect the intramuscular pH and Pi levels, compared with wearing CG applying 8 or 25 mmHg. The finding suggested that optimal pressure intensity would exist to obtain beneficial effects of wearing CG. However, similar studies using different levels of CG pressures did not show changes in running performance [12] or oxygen uptake [13]. Taken together, appropriate levels of compressive pressure to improve running performance remain unclear.

Prolonged running, such as marathon, elicits marked muscle damage and inflammation, leading to impaired muscular function [14–16]. Del Coso et al. [16] reported that a decrease in running velocity over a marathon was significantly correlated with an increase in blood myoglobin (Mb) concentration immediately after exercise. Considering that sustained external pressure while wearing CG attenuates mechanical stress by reducing muscle oscillation [17], the use of CG during exercise might attenuate increases in muscle damage markers and inflammatory cytokines. Another mechanism underlying effectiveness of wearing CG may be improved venous return, resulting from assisted muscle pump action by the garments [18]. The augmented muscle pump action by wearing CG would be more advantageous when exercise intensity is relatively lower, because muscle pump action is dependent on exercise intensity. In addition, positive effect of wearing CG is suggested to be observed during prolonged exercise with accumulated fatigue [1]. However, most of these previous studies have focused on the effectiveness of wearing CG on cardiovascular and metabolic variables during maximal or intensive endurance exercise lasting less than 60 min [1,3,7,12,13,18–20]. Therefore, little information is available on whether wearing CG during prolonged running (> 60 min) affects exercise-induced changes in muscle damage markers and the inflammatory responses.

The aim of this study was to investigate the effect of wearing lower-body CG at three different pressure levels (30 mmHg, 15 mmHg, below 5 mmHg) during 120 min of running on exercise performance, muscle damage markers, and the inflammatory response. Based on previous studies [3,10], it was hypothesized that wearing CG exerting medium (approximately 15 mmHg) pressure would attenuate the decrease in exercise performance and increases in muscle damage and inflammatory markers during prolonged running.

## Methods

### Participants

Eight male (mean ± standard deviation: age, 23.4 ± 2.4 years; height, 170.1 ± 2.2 cm; body mass, 62.3 ± 3.3 kg; body mass index, 21.9 ± 1.6 kg/m^2^; VO_2_max, 50.6 ± 4.1 ml/kg/min) participated in the present study. Two of ten subjects were not able to complete 120 min of exercise (exhausted at 90 min). All participants were physically active (i.e., exercising at least 1 day/week) but not well-trained athletes. They had several years of sports participation experience. Exclusion criteria included a history of inflammatory condition and musculoskeletal disorders. Smokers and individuals taking antioxidant supplements were also excluded. Participants were instructed to maintain their normal diet and physical activity level throughout the experimental period. They were also asked to refrain from strenuous activity for at least 72 h prior to testing. All participants gave informed consent after being informed of the purpose and risks associated with the present study. This study was approved by the Ethics Committee of the Ritsumeikan University, Japan.

### Experimental procedure

All participants visited the laboratory four times over the experimental period. On the first day, they completed an incremental running test on a treadmill (Valiant; Lode B.V., Groningen, Netherlands) to determine individual VO_2_max. The initial velocity was set as 4 km/h for 3 min, and velocity was increased by 2 km/h every 3 min. All participants were required to walk both the 4km/h and 6km/h stages, and they started running from 8km/h stage. After completing above three stages of submaximal running (9 min after exercise onset), running velocity was increased by 0.6 km/h every minute until exhaustion. The treadmill gradient was 7% throughout the test. Respiratory gases were collected and analyzed using an automatic gas analyzer (AE300S; Minato Medical Science, Tokyo, Japan). Participants also engaged in sufficient practice of appropriate procedures for the counter-movement jump (CMJ) test and reviewed the subjective rating scale of the measurements for familiarization.

From the second to fourth visits, participants performed three exercise trials with overnight fast in a random order at the same time of the day. The exercise consisted of 120 min of uphill running (slope: 7%) on a treadmill (Elevation series E95Ta; Life Fitness Corp., Tokyo, Japan) at 60% of VO_2_max while wearing one of three different types of garment, separated with at least 4 weeks among trials to eliminate repeated-bout effect. The average running velocity was 6.6 ± 0.5 km/h. Uphill running was selected to induce greater metabolic response under relatively lower running velocity. All participants maintained running throughout 120 min of exercise. The present exercise protocol was determined on the basis of a pilot experiment.

Custom-made garments were utilized to match pressure levels among all participants because interindividual differences in thigh and calf circumferences were reported to produce different pressure levels for working muscles, even if the garment is the same size [5]. In the present study, appropriate size of the garment was medium for all participants. However, we prepared several types of garment (different width) for each trial (five garments for the HIGH and MED trials, respectively, three garments for the CON trial), and a garment from several types was selected individually to ensure equal pressure levels among all participants. The compression levels at the thigh and calf were manipulated to match about 30 mmHg for HIGH trial, about 15 mmHg for MED trial, and < 5 mmHg for CON trial, respectively. Accordingly, the exercise trials consisted of 1) wearing lower-body CG exerting high level of pressure (approximately 30 mmHg) [HIGH]; 2) wearing lower-body CG exerting medium level of pressure (approximately 15 mmHg) [MED]; and 3) wearing a lower-body garment without specific pressure level (< 5 mmHg) [CON]. Each garment covered the area from the waist to the ankle and was custom-made by a sportswear manufacturer (DESCENTE Ltd., Osaka, Japan). Prior to the experiment, the pressure levels were determined using an air-packed sensor (AMI3037-2; AMI Techno, Tokyo, Japan). The sensor was placed between the skin and the garment at the thigh (50% between the greater trochanter and patellar tendon) and calf (30% between the patellar tendon and lateral malleolus). Participants were asked to maintain a standing position for 15 s while the pressure levels were recorded, and mean values were calculated during 15 s. The compression levels of each garment are presented in Table 1.

**Table 1:** Compression applied by CG at the thigh and calf.

	Compression at thigh	Compression at calf
	(mmHg)	(mmHg)
HIGH	26.9 ± 3.3	29.2 ± 3.8
	[22.6–32.8]	[24.5–37.7]
MED	16.1 ± 2.0	17.9 ± 3.5
	[14.3–20.1]	[14.9–25.5]
CON	4.4 ± 1.2	3.0 ± 1.6
	[2.5–5.8]	[0.7–4.8]

Values are means ± SD. The values in parentheses indicate minimal and maximal pressure levels among all participants.

Participants wore identical upper body shirts, socks, and shoes during the 120 min exercise and 60 min rest periods among trials. Time course changes in jump performance, rating of perceived exertion (RPE), HR, and blood variables were compared among the three trials.

### Jump performance

CMJ height was evaluated before exercise (after 20 min of rest) and immediately after exercise, as an indication of maximal power output by the lower-limb muscles. We used CMJ height rather than maximal isometric strength as an indication of muscle function, as a decrease in CMJ height is reported to be related to muscle damage and decreased performance during a marathon race [14]. All participants performed the CMJ on a jump mat (Multi jump tester; DKH Corp., Tokyo, Japan) connected to a computer. They were instructed to jump as high as possible while placing their hands on the lumbar area to eliminate any upper-limb effect. The flight and contact time during the vertical jump were recorded. Each jump test was repeated twice, and the highest CMJ height value was used for analysis in each case. The intraclass correlation coefficient between jumps was 0.99. CMJ height was calculated from flight time using the following formula:

Jump height (cm) = 1/8 (flight time)^2 × the gravity constant (= 9.81 m/s^2)

### Heart rate and rating of perceived exertion

HR, and the RPE values for respiration and legs were monitored every 10 min during 120 min of uphill running. HR was measured continuously using a wireless HR monitor (RCX5; Polar, Tokyo, Japan). The RPE values for respiration and legs were recorded using the modified Borg scale ranging from 0 (*nothing at all*) to 10 (*maximal exertion*) [21]. Participants were instructed to answer magnitude of strains for legs and respiration during running (“how do you feel the levels of fatigue for leg muscles and respiration?”) to assess RPE values.

### Blood sampling and analysis

Blood samples were collected from antecubital vein before exercise (after 20 min of rest), 60 min during exercise, and immediately and 1 h after exercise. Blood samples were used to measure blood glucose, lactate, serum creatine kinase (CK), Mb, free fatty acids (FFA) and glycerol, cortisol, and plasma interleukin-6 (IL-6) concentrations. In addition to indirect muscle damage markers (CK and Mb) and inflammatory cytokine (IL-6), serum FFA, glycerol and cortisol concentrations were assessed to evaluate metabolic responses to 120 min of running. Serum and plasma samples were obtained by centrifugation (3,000 rpm, 10 min, and 4°C) and stored at −60°C until analysis. Blood glucose and lactate concentrations were measured using an automatic glucose analyzer (Free style, Nipro Corp., Osaka, Japan) and a lactate analyzer (Lactate Pro; Arkray Inc., Kyoto, Japan), respectively. Serum CK, Mb, FFA, and cortisol concentrations were measured at the SRL Clinical Laboratory (Tokyo, Japan). Serum glycerol concentrations were determined in duplicate using a commercially available kit (Cayman Chemical Co., Ann Arbor, MI, USA). Plasma IL-6 concentrations were assayed with an enzyme-linked immunosorbent assay kit (R&D Systems, Minneapolis, MN, USA). The intra-assay coefficients of variation for each measurement were: 2.7% for CK, 2.2% for Mb, 1.5% for FFA, 2.5% for glycerol, 2.9% for cortisol, and 4.4% for IL-6.

### Statistical analysis

Data are expressed as means ± standard deviation. Time course changes in jump performance, RPE, HR, and the blood variables were initially analyzed using a two-way analysis of variance (Trial x Time) with repeated measures. When the ANOVA revealed a significant interaction or main effect, the Tukey–Kramer *post-hoc* test was used to assess the difference. Where appropriate, partial eta-squared (η^2^) was used to quantify the effect size of ANOVA. A *P*-value < 0.05 was considered significant.

## Results

**Fig 1:**
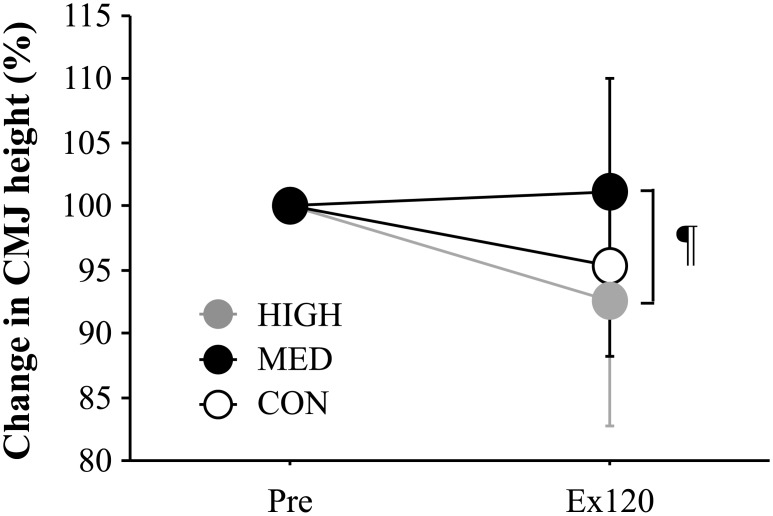
Changes in CMJ height. Values are mean ± standard deviation. ¶; *P* < 0.05 between MED and HIGH. Ex120; immediately after exercise.

The relative changes in CMJ height are presented in Fig 1.

A significant interaction (trial × time, *P* = 0.04, η^2^ = 0.368) and main effect for trial (*P* = 0.04, η^2^ = 0.368) were detected, whereas no significant main effect for time was observed (*P* = 0.172). Immediately after exercise, CMJ height was significantly higher in the MED trial (101.1 ± 3.2%) compared with that in the HIGH trial (92.6 ± 3.4%, *P* = 0.04, η^2^ = 0.368).

**Fig 2:**
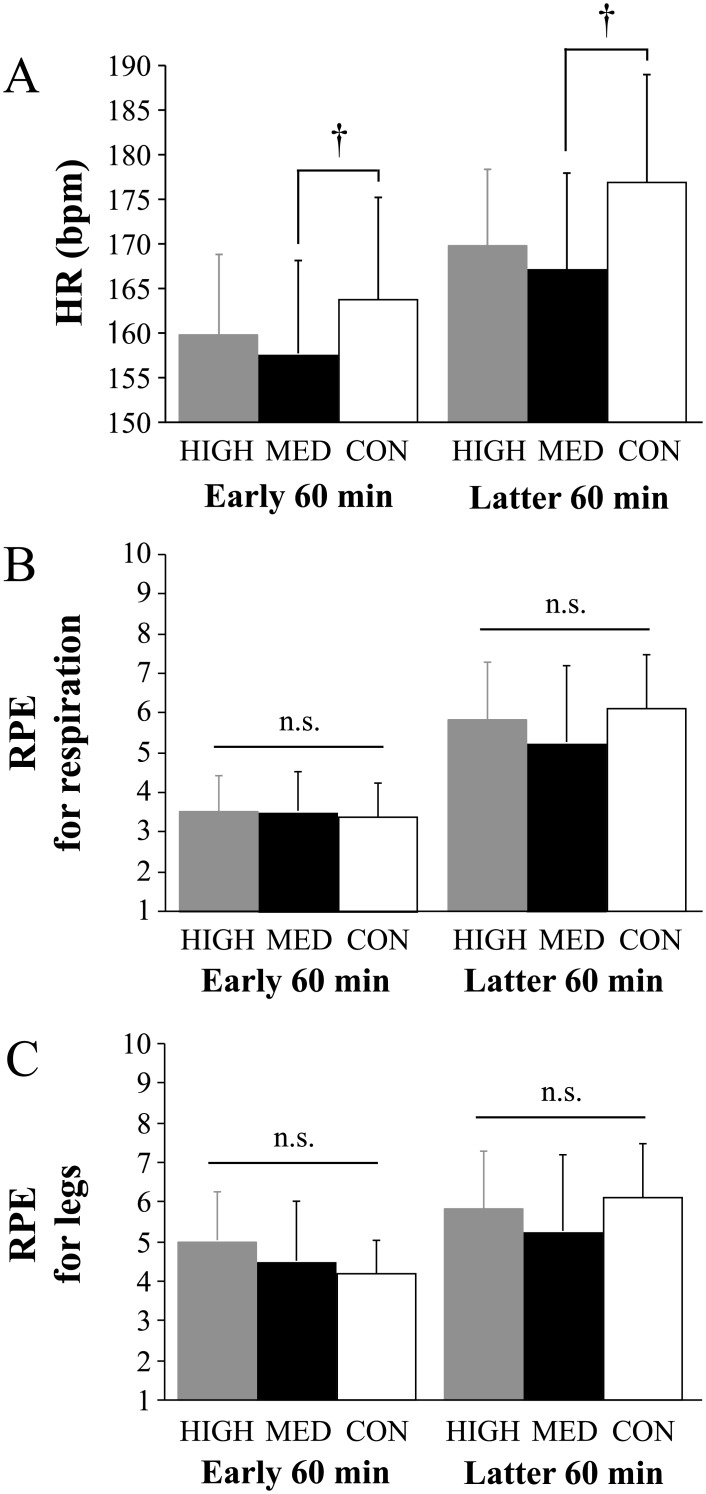
Mean HR values (A), mean RPE values for respiration (B) and mean RPE values for legs (C) during 120 min of running. Values are mean ± standard deviation. †; *P* < 0.05 between MED and CON.

Fig 2A shows mean HR for early (0–60 min) and latter (60–120 min) halves of the exercise. HR increased gradually during exercise in all trials (main effect for time: *P* < 0.0001, η^2^ = 0.844). Mean HR were significantly lower in the MED trial (0–60 min: 158 ± 4 bpm, 60–120 min: 167 ± 4 bpm) than those in the CON trial (0–60 min: 164 ± 4 bpm, 60–120 min: 177 ± 4 bpm, *P* = 0.01, η^2^ = 0.479 for mean HR for early half and η^2^ = 0.476 for mean HR for latter half), respectively. Significant main effect for time was observed for RPE of respiration and legs (*P* < 0.0001, η^2^ = 0.793 for RPE of respiration and η^2^ = 0.916 for RPE of legs). No significant differences were observed in mean RPE of respiration or legs for early (0–60 min) and latter (60–120 min) halves of the exercise (*P* > 0.05, Fig 2B and 2C).

Table 2 presents the changes in blood glucose and lactate, as well as serum CK and Mb concentrations. Significant main effect for time was observed in all variables (*P* < 0.05). However, no significant interaction (trial × time) or main effect for trial were observed.

**Table 2:** Blood glucose, lactate and serum CK and Mb concentrations.

		Pre	Ex60	Ex120	Post60
Glucose (mg/dL)	HIGH	94 ± 6	85 ± 11	79 ± 16 *	75 ± 11 *
	MED	90 ± 6	84 ± 4	79 ± 10 *	77 ± 6 *
	CON	90 ± 8	89 ± 8	79 ± 18 *	74 ± 12 *
Lactate (mmol/L)	HIGH	1.8 ± 0.9	2.1 ± 1.1	2.9 ± 1.4 *	2.0 ± 0.3 *
	MED	1.6 ± 0.6	2.0 ± 1.1	2.3 ± 1.4 *	2.1 ± 1.0 *
	CON	1.2 ± 0.3	1.7 ± 0.8	2.2 ± 0.8 *	2.0 ± 0.4 *
CK (U/L)	HIGH	142 ± 46	170 ± 51 *	196 ± 63 *	193 ± 58 *
	MED	144 ± 57	172 ± 68 *	198 ± 73 *	195 ± 68 *
	CON	191 ± 177	225 ± 195 *	259 ± 118 *	252 ± 189 *
Mb (ng/mL)	HIGH	31 ± 8	55 ± 21	72 ± 31 *	106 ± 36 *
	MED	32 ± 13	59 ± 29 *	82 ± 30 *	106 ± 32 *
	CON	36 ± 21	66 ± 49	92 ± 18 *	131 ± 61 *

Values are means ± SD.

*; P < 0.05 vs. Pre. Ex60; 60 min during exercise. Ex120; immediately after exercise. Post60; 60 min after exercise.

**Fig 3:**
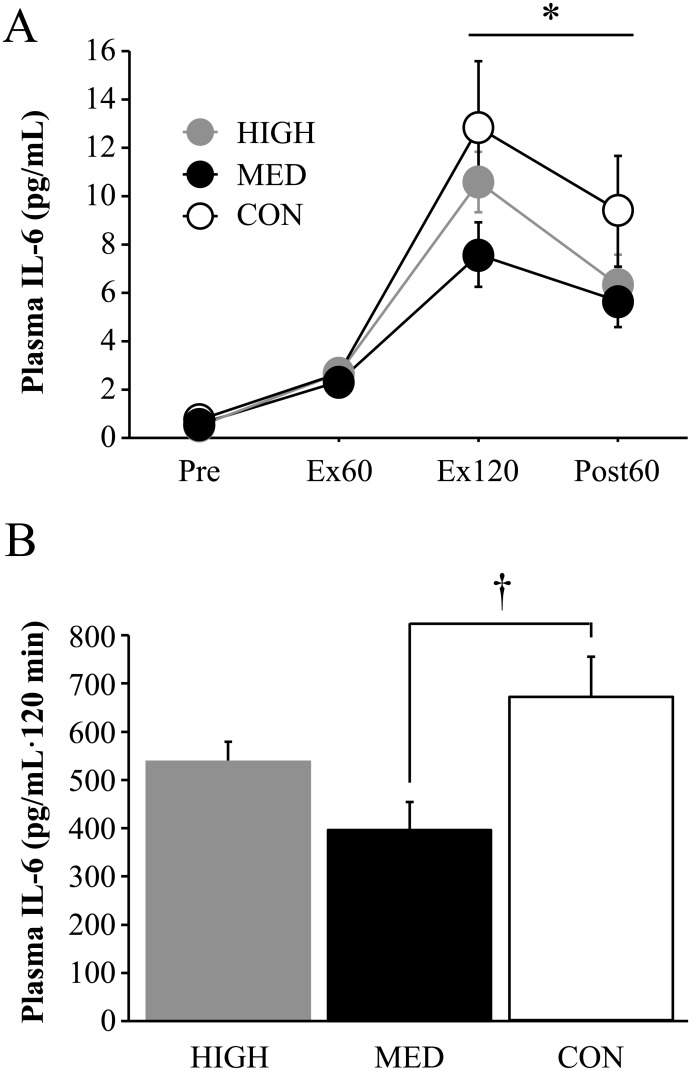
Plasma interleukin (IL)-6 concentrations (A) and area under the curve (AUC) during 120 min of exercise. Values are mean ± standard deviation. *; *P* < 0.05 vs. Pre. †; *P* < 0.05 between MED and CON. Ex60; 60 min during exercise. Ex120; immediately after exercise. Post60; 60 min after exercise.

Plasma IL-6 concentrations increased significantly during the exercise and post-exercise periods in all trials (main effect for time: *P* < 0.0001, η^2^ = 0.849), but no significant interaction (*P* > 0.05) or main effect for trial was detected (*P* > 0.05, η^2^ = 0.229, Fig 3A). However, the area under the curve (AUC) for IL-6 during 120 min exercise period was significantly lower in the MED trial (397 ± 58 pg/ml·120 min) compared with that in the CON trial (670 ± 86 pg/ml·120 min, *P* = 0.04, η^2^ = 0.454, Fig 3B). No significant difference was observed between the HIGH and CON trials.

## Discussion

The major finding of the present study was that the MED trial showed a significantly lower exercise-induced decrease in CMJ height compared with that of the HIGH trial and a smaller increase in HR compared with that in the CON trial. Furthermore, the increased plasma IL-6 concentration during 120 min of running was also impaired in the MED trial than in the CON trial. These findings suggest that optimal pressure (approximately 15 mmHg) exists for decrease in exercise-induced fatigue during prolonged running.

A unique point of the present study was preparation of several types of custom-made garments to match pressure levels (approximately 15 and 30 mmHg) among participants, because no consensus has been reached about the anti-fatiguing effect of CG, probably due to differences in the compressive pressures applied among studies [22] and participants [5]. Furthermore, we attempted to determine the influences of CG exerting different pressure levels on exercise-induced decrease in jump performance. As a result, an exercise-induced decrease in CMJ height was significantly greater in the HIGH trial than in the MED trial. Decreased jump height following prolonged running would be caused by several factors, including impaired neuromuscular function [23], accumulated metabolites [24] and muscle damage [14]. In particular, previous studies revealed that decreases in jump height and running velocity closely related to exercise-induced increases in Mb and CK concentrations immediately after prolonged running [14]. Thus, greater jump height immediately after exercise, as shown in the MED trial, may reflect maintained lower-limb muscle function during prolonged running. A possible mechanism for improved jump performance may be augmented removal of metabolites (e.g., H^+^ and inorganic phosphate) in working muscle because the reduction in intramuscular pH inhibits muscle contractile function (e.g., shortening velocity) [25]. This notion is supported by previous findings revealing that adequate external pressure assists muscle pump action and improves peripheral circulation, leading to enhanced removal of H^+^ and inorganic phosphate from working muscle [11,26]. Notably, the effect of lower-body CG on the exercise-induced decrease in jump height was not dependent on the pressure level applied since the post-exercise jump height was significantly lower in the HIGH trial compared with that in the MED trial. Although a high pressure CG would theoretically augment venous return from working muscle, excessive pressure exerted by CG (30–40 mmHg) significantly attenuates local blood flow [10,27]. The reduced local blood flow associated with the strong external pressure applied by CG would aggravate the accumulation of muscle metabolites [28,29]. Accordingly, impaired jump height immediately after the exercise period in the HIGH trial might be explained by the accumulation of intramuscular metabolites under lower blood flow. Future applications evaluating local blood flow would clarify this hypothesis.

Exercise-induced decrease in CMJ height was significantly correlated with increases in CK and Mb concentrations immediately after prolonged exercise (triathlon race) [14]. In the present study, no differences in CK or Mb concentrations were observed among the three trials. In contrast, the exercise-induced increase in IL-6 concentration (evaluated by AUC) was significantly lower in the MED trial compared with that in the CON trial. The finding suggests that CG with medium pressure level attenuates exercise-induced inflammation. However, because blood samples were collected during relatively initial phase (60 min) of post-exercise period, we are not able to conclude the influence of wearing CG on muscle damage and inflammation after 60 min following the exercise. Future study is necessary to determine the changes in muscle damage markers and inflammatory cytokines during longer period after exercise.

A large amount of evidence indicates that HR during exercise is not affected by wearing CG [1–3,7,18]. However, the MED trial significantly attenuated the exercise-induced increase in HR. These inconsistent observations among studies may be associated with differences in exercise intensities, as previous studies required relatively high or maximal efforts during exercise, whereas the exercise intensity in the present study was moderate (60% of VO_2_max). Furthermore, mean running velocity was low (6.6 ± 0.1 km/h) because we utilized uphill running. MacRae et al. [18] pointed out that a plausible factor for lower HR during wearing CG was augmented stroke volume associated with increased venous return. Considering that venous return would be augmented depending on exercise intensity, the influence of CG on hemodynamics is supposed to be minor during intensive exercise [3]. In fact, the wearing lower-body CG during 6 km/h treadmill running significantly attenuated the exercise-induced increase in HR, but not during 10 km/h or at 85% of VO_2_max running [30]. Besides, the majority of previous studies used CG that provided gradually decreasing pressure levels from the distal part (ankle) to the proximal part (thigh) to enhance venous return. However, the pressures exerted between the thigh and calf were not different in the present study (Table 1), suggesting that the pressure levels were uniform across the lower-limb muscles. Therefore, wearing lower-body CG exerting medium pressure (approximately 15 mmHg) at both the thigh and calf is thought to be beneficial for reducing the exercise-induced increase in HR.

Some limitations need to be considered carefully in the present study. First, the number of participants was relatively small. This is because several types of custom-made garments with different width were prepared for each size of garments (five garments for the HIGH and MED trials, respectively, three garments for the CON trial) to match pressure levels applied among participants. However, further experiment with larger sample size would be informative to assist the present findings. Second, we were unable to identify changes in maximal muscular strength, hemodynamics, cardiovascular response, intramuscular metabolites in response to prolonged running. Therefore, how wearing CG attenuated exercise-induced decrease in jump performance still needs further determination. However, the present findings provide novel insight for the importance of compressive pressure in performance enhancement. Since prolonged running under relatively lower running velocity was selected in the current study, the findings would be specially applicable for recreational or amateur endurance runners.

## Conclusion

Wearing lower-body CG exerting 15 mmHg on the thigh and calf attenuated decrease in jump performance compared with wearing lower-body CG exerting 30 mmHg. Furthermore, exercise-induced increases in HR and IL-6 concentration with CG were significantly smaller in CG exerting 15 mmHg than in garment with exerting < 5 mmHg.
